# Characterization of Systemic and Mucosal Humoral Immune Responses to an Adjuvanted Intranasal SARS-CoV-2 Protein Subunit Vaccine Candidate in Mice

**DOI:** 10.3390/vaccines11010030

**Published:** 2022-12-23

**Authors:** Mariam Maltseva, Yannick Galipeau, Tyler M. Renner, Lise Deschatelets, Yves Durocher, Bassel Akache, Marc-André Langlois

**Affiliations:** 1 Department of Biochemistry, Microbiology & Immunology, Faculty of Medicine, University of Ottawa, Ottawa, ON K1H 8M5, Canada; 2 National Research Council Canada, Human Health Therapeutics, Ottawa, ON K1A 0R6, Canada; 3 Ottawa Center for Infection, Immunity and Inflammation (CI3), Ottawa, ON K1H 8N5, Canada

**Keywords:** COVID-19, SARS-CoV-2, subunit vaccine, spike, receptor binding domain, mucosal immunity, AMVAD, intranasal vaccine

## Abstract

Continuous viral evolution of SARS-CoV-2 has resulted in variants capable of immune evasion, vaccine breakthrough infections and increased transmissibility. New vaccines that invoke mucosal immunity may provide a solution to reducing virus transmission. Here, we evaluated the immunogenicity of intranasally administered subunit protein vaccines composed of a stabilized SARS-CoV-2 spike trimer or the receptor binding domain (RBD) adjuvanted with either cholera toxin (CT) or an archaeal lipid mucosal adjuvant (AMVAD). We show robust induction of immunoglobulin (Ig) G and IgA responses in plasma, nasal wash and bronchoalveolar lavage in mice only when adjuvant is used in the vaccine formulation. While the AMVAD adjuvant was more effective at inducing systemic antibodies against the RBD antigen than CT, CT was generally more effective at inducing overall higher IgA and IgG titers against the spike antigen in both systemic and mucosal compartments. Furthermore, vaccination with adjuvanted spike led to superior mucosal IgA responses than with the RBD antigen and produced broadly targeting neutralizing plasma antibodies against ancestral, Delta and Omicron variants in vitro; whereas adjuvanted RBD elicited a narrower antibody response with neutralizing activity only against ancestral and Delta variants. Our study demonstrates that intranasal administration of an adjuvanted protein subunit vaccine in immunologically naïve mice induced both systemic and mucosal neutralizing antibody responses that were most effective at neutralizing SARS-CoV-2 variants when the trimeric spike was used as an antigen compared to RBD.

## 1. Introduction

The ongoing emergence of highly transmissible SARS-CoV-2 variants of concern (VOCs) have significantly hampered vaccine effectiveness against infection, COVID-19 disease and transmission [1,2,3]. Reduced vaccine effectiveness can also be attributed to immune evasion by VOCs, waning immunity following prolonged periods post vaccination or natural infection and poor induction of mucosal immunity [4,5]. Currently, approved intramuscular (IM) vaccines induce robust systemic immunity which protects against severe disease yet leaves the individual vulnerable to infection and reinfection at the primary point of viral entry at the upper respiratory tract (URT) [6,7]. Indeed, breakthrough infections with viral shedding, with both Delta and Omicron variants, have been reported in fully vaccinated individuals, highlighting the possibility of onward viral transmission despite vaccination [8,9,10]. While vaccine boosting has significantly increased vaccine effectiveness against severe disease and hospitalization, continued viral evolution and emergence of highly transmissible VOCs highlight the need for revaluating our vaccine strategy, due to the shortcomings of currently available vaccines in reducing transmission [4]. In the effort to curtail the COVID-19 pandemic, next generation vaccines must address immune evasion and provide improved cross-protection against rapidly emerging SARS-CoV-2 variants at the point of entry in order to reduce viral transmission.

Several groups have been evaluating transmission blocking strategies that target SARS-CoV-2 entry. The spike protein of SARS-CoV-2 is the primary antigenic target of currently approved vaccines due to its high immunogenicity, abundant viral surface expression and ability to elicit both humoral and cell-mediated responses [11,12,13,14,15,16,17,18]. The spike protein also demonstrates effective mucosal and systemic induction of humoral and cell-mediated immunity through oral and intranasal vaccination in various animal models [19,20,21,22,23,24,25,26,27,28,29,30]. Notably, a majority of these vaccine candidates are based on an adenoviral platform which expresses the spike trimer protein of the parental strain of SARS-CoV-2. Indeed, these groups demonstrated superior protection with intranasal vaccination relative to intramuscular immunization upon subsequent viral challenge, in both the upper and lower respiratory tract, against the ancestral strain and VOCs [19,21,23,24,28]. These strategies targeting the respiratory mucosa reveal effective protection against disease and reduced viral shedding in mice, hamsters and non-human primates [19,20,21,23,24,25,30,31]. While spike trimer formulations are more commonplace, vaccines targeting the receptor binding domain (RBD) of the spike have been proposed, given its direct interaction with the cognate entry receptor, angiotensin-converting enzyme 2 (ACE2) [32]. Additionally, many potent RBD-specific neutralizing antibodies have been characterized from convalescent and vaccinated individuals [13,16,17,33,34,35,36]. While the immunogenicity of these antigens has been extensively characterized following intramuscular immunization in pre-clinical animal models, their relative immunogenicity as intranasal vaccine targets against emerging VOC remain unclear. To our knowledge, this is the first study directly comparing the systemic and mucosal humoral responses following the intranasal administration of SARS-CoV-2 spike and RBD antigens in mice.

Adjuvants are often essential for directing antigen-specific immune responses and overcoming weak immunogenicity of protein subunit vaccines. Due to adjuvants’ intrinsic immunomodulatory properties, their combined administration with an antigen elicits superior and long-lasting immune responses while overcoming natural tolerances of mucosal compartments to antigens administered alone [7,37,38]. Indeed, mucosal adjuvants need to overcome the harsh conditions of mucosal tissues (e.g., low pH, continuous mucociliary clearance and local enzymes) and rapidly permeate mucus layers in order to induce efficacious responses and establish immune memory [39,40]. Cholera toxin (CT) is produced by *Vibrio cholerae* and its combined intranasal administration with an antigen has been shown to elicit potent antigen-specific IgA responses in the URT and T cell-mediated responses in animal models [41,42,43,44,45]. Although CT is among the most effective mucosal adjuvants in animal models, due to safety concerns linked to toxin-mediated neuroinflammation that correlate with Bell’s palsy, its use as an adjuvant has been precluded from the clinic [43,46,47]. In contrast, the archaeal polar lipid mucosal vaccine adjuvant and delivery (AMVAD) system used in this study is a self-adjuvating mucosal vaccine delivery system with an established preclinical safety profile that does not require any additional immunostimulants and can withstand a broad range of pH [48,49,50,51]. AMVAD is characterized by its polar lipid and large spherical structure which aggregates in a grape-like formation, thereby encapsulating the antigen [48,52]. Various antigens and formulations of lipid archaeosome-based adjuvants administered intramuscularly have been evaluated in vivo and were shown to elicit robust cell-mediated and humoral responses through recruitment and activation of macrophages and dendritic cells [53,54,55,56]. In turn, while intranasal immunization using these specific adjuvants is less explored, Patel et al. demonstrate that intranasally administered ovalbumin adjuvanted with AMVAD induced robust mucosal and systemic ovalbumin IgA responses in mice [48].

In this study, we investigated the immunogenicity of an intranasally administered protein subunit vaccine expressing the stabilized SARS-CoV-2 full spike trimer ectodomain or RBD adjuvanted with either CT or AMVAD. We show robust induction of IgG and IgA responses in plasma, nasal wash and bronchoalveolar lavage fluid (BALF) in mice. We observed induction of mucosal and systemic neutralizing IgA and IgG responses. The observed systemic responses were of superior or similar neutralization capacity compared to serum antibodies elicited in humans by SARS-CoV-2 infection or two doses of mRNA vaccine, respectively. Mucosal IgA and systemic IgG antibody titers correlated with neutralizing antibody levels in BALF and plasma, respectively. Given the need for the development of next generation vaccines that target SARS-CoV-2 at the primary site of infection, our findings highlight the immunogenicity of an adjuvanted subunit vaccine platform in stimulating both mucosal and systemic humoral responses in mice and eliciting neutralizing antibodies against emerging SARS-CoV-2 variants.

## 2. Materials and Methods

### 2.1. Protein Production

RBD protein was produced in HEK 293F cells and purified. Briefly, a plasmid generously provided by Florian Krammer (Mount Sinai, New York, NY, USA) encoding the Wuhan-Hu-1 RBD (MN908947) sequence coding for the amino acid 319–541 and fused with the N-terminal SARS-CoV-2 spike secretory signal and a C-terminal hexa-histidine tag was transfected into 293F cells cultivated in Freestyle 293 expression media (Thermo Fisher, #12338018) at 37 °C, 7% CO_2_, while shaking (125 rpm). A total of 600 million cells resuspended in 200 mL were transfected with 200 μg of plasmid using ExpiFectamine (Thermo Fisher, 14525, Waltham, MA, USA). Three days post-transfection, cell supernatant was harvested by centrifugation (4000× *g* for 20 min at 4 °C) and filtered through a low binding 0.22 μm Stericup vacuum filter (Millipore Sigma, S2GPU10RE, Burlington, MA, USA). The filtered supernatant was incubated for 2 h at room temperature with 6 mL of Ni-NTA resin (Qiagen, 30210, Hilden, Germany). The column containing the mix of supernatant and resin was washed four times with a washing buffer containing 20 mM imidazole, 300 mM NaCl and 57.5 mM of NaH_2_PO_4_·H_2_O. The RBD protein was then eluted with three column volumes of the elution buffer containing 234 mM of imidazole, 300 mM NaCl and 57.5 mM of NaH_2_PO_4_·H_2_O. The eluted solution was concentrated, and the buffer was replaced with PBS using a 10 kDa Amicon filter (Millipore Sigma, UFC901008). RBD protein integrity was verified by SDS-PAGE, aliquoted to minimize freeze–thaw cycles and stored at −80 °C. Prefusion trimer spike production details are published elsewhere [57].

### 2.2. Mice Immunization and Sample Collection

All experiments performed on mice were conducted in accordance with Ontario Animals for Research Act and were approved by the University of Ottawa Animal Ethics Committee (protocol BMI-3649). Female BALB/C mice (6–12 weeks) were obtained from Charles River Laboratories (Saint-Constant, Canada). Prior to each immunization, mice were anesthetized with isoflurane and vaccinated intranasally (25 μL per nare) with 20 μg RBD or spike protein admixed with cholera toxin (CT) (1 μg per dose) used here as a positive control (Sigma, Oakville, ON, Canada) or AMVAD mucosal adjuvant (400 μg per dose) on days 0 and 21. The AMVAD formulation was prepared with total polar lipids from *Methanobrevibacter smithii* and combined with antigen in a saline-based solution containing a final concentration of 15 mM CaCl_2_, as previously described [48,51]. Control groups include mice vaccinated with phosphate-buffered saline (PBS), protein alone (spike) and adjuvant alone (CT or AMVAD). Plasma was collected periodically throughout the study via submandibular vein and heart puncture at end point. Seven days post boost, bronchoalveolar lavage fluids (BALF) and nasal wash were collected to assess for mucosal humoral responses. Nasal wash and BALF samples were concentrated through 50 kDa Pierce Protein Concentrators column (Thermo Fisher Scientific, Waltham, MA, USA) according to the manufacturer’s instructions, with volumes normalized prior to concentration.

### 2.3. Indirect ELISA to Evaluate Anti-SARS-CoV-2 Immunoreactivity in Serum Samples (Serology)

The same SARS-CoV-2 trimer spike or RBD antigens that were administered for immunizations for used as coating antigens for serological assays. SARS-CoV-2 trimer spike or RBD were diluted in sterile 1X PBS (Wisent Bioproducts, 311-010-CL, St-Bruno, QC, Canada) to 4 μg/mL and coated onto 384-well Immuno plates (Thermo Fisher, #60372, Waltham, MA, USA) (10 μL/well) overnight at 4 °C. Plates were washed three times with 100 μL of PBS-T and blocked for 1 h with a blocking buffer (PBS-T + 3% non-fat milk powder, *w*/*v*) on a shaker at room temperature. Serum samples were diluted 1:100 (following first immunization) and (1:2000 following second immunization), BALF was diluted 4- to 30-fold and nasal wash was diluted 4-fold in dilution buffer (PBS-T + 1% non-fat milk powder, *w*/*v*). After blocking, plates were washed thrice with PBS-T, and followed by addition of 10 μL of the respective diluted samples. The plates were incubated for 2 h on a shaker at room temperature, washed thrice with PBS-T followed by the addition of 10 μL of the respective secondary-HRP antibody at specified dilutions: 1:4000 secondary IgG Anti-Mouse, Human ads-HRP (Southern Biotech, 1030-05, Birmingham, AL, USA) and 1:1000 secondary Anti-Mouse IgA-HRP (Southern Biotech, 1040-05, Birmingham, AL, USA). Plates were incubated for 1 h on a shaker, washed thrice with PBS-T followed by the addition with 10 μL of the SuperSignal ELISA Pico Chemiluminescent Substrate (Thermo Scientific, 37069, Waltham, MA, USA). Luminescence intensity was measured with BIO-TEK Synergy Neo2 plate reader for 20 ms/well at a read height of 1.0 mm. Wells filled with dilution buffer in place of sample accounted for background luminescence and were subtracted from the sample values.

### 2.4. Surrogate Neutralization ELISA (snELISA) Assay to Evaluate Neutralization Activity in Serum Samples

The described methodology was adapted from the surrogate neutralization ELISA assay, as shown in Colwill et al. and Demone et al., for the evaluation of the relative inhibition of neutralizing antibodies to spike or RBD protein from binding to soluble ACE2 [57,58]. Briefly, SARS-CoV-2 spike or RBD protein were diluted in sterile 1X PBS to 8 μg/mL and coated onto 384-well Immuno plates (12.5 μL/well) overnight at 4 °C. Plates were washed 3 times with PBS-T and blocked for 1 h while shaking. Plasma samples were 1:6 serially diluted in a dilution buffer while BALF was left undiluted, applied to wells (20 μL/well) and incubated for 2 h while shaking at room temperature. Plates were washed thrice, followed by the addition of biotinylated ACE2, as shown in Abe et al. [59], diluted to 0.35 ng/μL in a dilution buffer (20 μL/well). Plates were washed thrice with PBS-T followed by the addition of 20 μL per well of Streptavidin–Peroxidase polymer (Sigma, S2438, Oakville, ON, Canada) diluted in dilution buffer to 1.25 ng/μL. Following 1 h incubation, plates were washed thrice with PBS-T and freshly diluted SuperSignal ELISA Pico Chemiluminescent Substrate (mixed 1:1 ratio and diluted in equal volume with dH_2_O, (*V*:*V*)) was applied (20 μL/well). Following 5 min of incubation while shaking, luminescence intensity was measured with BIO-TEK Synergy Neo2 plate reader for 20 ms/well at a read height of 1.0 mm. To assess the maximum binding signal, control wells were filled with a dilution buffer in place of serum followed by the addition of ACE2, as previously. Relative percent inhibition was calculated as follows:

$$\%\;Inhibition=\left(1-\frac{average\;mean\;of\;serum\;sample}{average\;mean\;of\;maxium\;signal}\right)\times 100$$

Serum dilution resulting in a 50% inhibition (half-maximal inhibitory dilution (ID_50_)) of spike or RBD protein from binding ACE2 receptor was determined using 4-parameter fitting with GraphPad Prism 9.1.2 software. When no neutralization activity was observed, the ID_50_ of the sample was arbitrarily set as half of the lowest dilution.

### 2.5. Patient Samples, Collection and Ethics Approval

Use of human samples for this study was approved by the University of Ottawa Ethics Review Board: Certificates H-04-20-5727, H-04-21-6643 and H-07-20-6009. The negative sample was taken from an individual with no history of SARS-CoV-2 infection and tested with PCR. Pooled negative samples were obtained from individuals negative for SARS-CoV-2 as tested by PCR and serology assay. Pooled serum samples were obtained from convalescent (2–16 weeks post-infection) or vaccinated patients (2–4 weeks post-vaccination) enrolled in surveillance studies from different research studies post-2019. Samples were collected using standard phlebotomy procedures. Samples were de-identified and held at 4 °C for short-term handling and testing at University of Ottawa CL2+ biocontainment facility. All research was performed in accordance with current guidelines and regulations.

## 3. Results

### 3.1. Intranasal Administration of Adjuvanted RBD and Spike Antigens Induces Robust Humoral Systemic Responses in Mice

**Figure 1:**
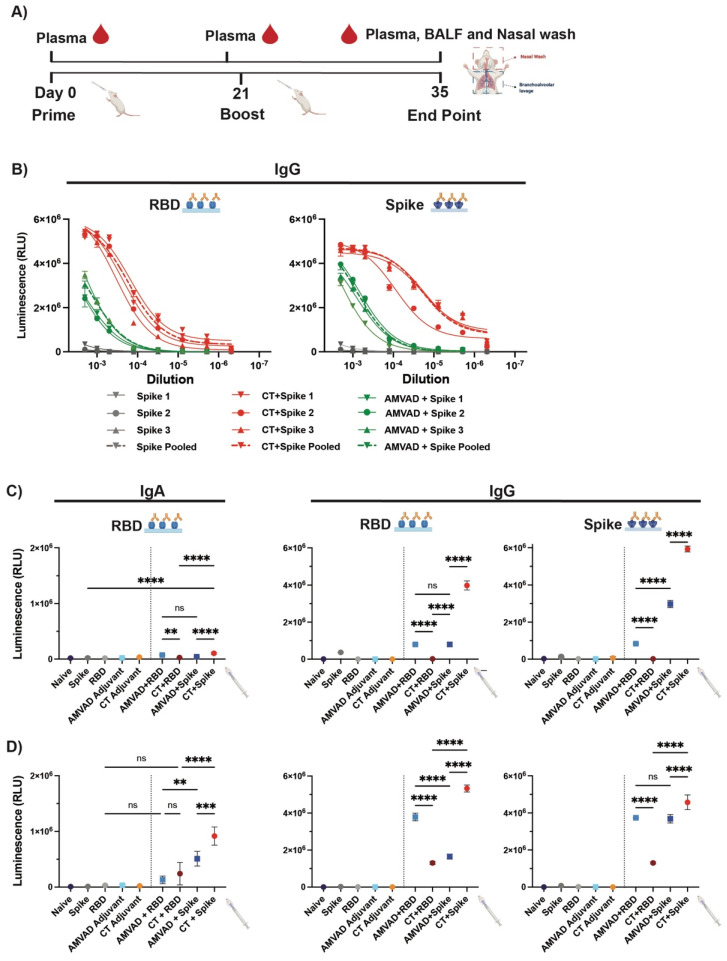
Humoral systemic responses from intranasal administration with adjuvanted RBD or spike antigens. (**A**) Experimental design: Balb/c mice were intranasally immunized with RBD or spike protein adjuvanted with CT or AMVAD mucosal adjuvant on days 0 and 21. Control groups include mice vaccinated with phosphate-buffered saline (PBS), protein alone (RBD or spike) and adjuvant alone (CT or AMVAD). Seven days post-boost, plasma, bronchoalveolar lavage fluids (BALF) and nasal wash were collected to assess humoral responses. (**B**) Evaluation of RBD- and spike-specific IgG titers in plasma of individual mice vs. pooled plasma collected at end point. (**C**) Measurement of RBD- and spike-specific IgA and IgG responses in plasma collected after first dose (day 20) (1/100 dilution) and (**D**) after second dose (day 35) (1/2000 dilution), analyzed by an indirect ELISA. Statistical significance was calculated by one-way ANOVA followed by Tukey correction; n.s.: no statistical difference; ** *p* ≤ 0.01, *** *p* ≤ 0.001, **** *p* ≤ 0.0001. Individual mice samples were pooled and analyzed in triplicate.

To assess immunogenicity of the intranasal subunit protein vaccine platform, we compared the ability of a stabilized prefusion trimeric spike or the RBD in eliciting mucosal and systemic antibody responses. Given that intranasally administered protein subunit vaccines have been shown to be poorly immunogenic on their own, we additionally assessed two different adjuvants: AMVAD and CT. Balb/c mice (n = 3/group) were immunized with controls (phosphate-buffered saline (PBS), soluble full spike trimer ectodomain, RBD, AMVAD or CT alone) or protein adjuvanted vaccine combinations (AMVAD + RBD, CT + RBD, AMVAD + spike, CT + spike) on day 0 and 21 (Figure 1A). Due to the limited amount of sample volumes obtained during the study and the numerous analyses we wanted to conduct, samples from immunized mice in each group were pooled together after confirming that the antibody responses in individual mice were similar. Figure 1B shows RBD or spike-specific IgG titers at various dilutions of three individual mice relative to pooled plasma samples, highlighting highly consistent seroconversion and antibody levels between mice within each group. Systemic antigen-specific IgG and IgA responses in plasma were measured after prime (day 20) (Figure 1C) or boost (day 35) (Figure 1D) immunization by enzyme-linked immunosorbent assay (ELISA) against RBD or spike. Following a single immunization, we show that there was poor induction of IgA antibodies with controls, including unadjuvanted antigens, as expected. In contrast, adjuvanted spike protein with AMVAD or CT elicited robust IgG titers in plasma (Figure 1C) that were significantly greater than with the adjuvanted RBD vaccine formulation after a single dose. As expected, we observed similar levels of RBD- and spike-directed IgG antibodies in mice immunized with the AMVAD + RBD vaccine formulation, highlighting that antibodies generated against RBD can access their epitopes on the trimeric spike. Following boosting, we observed a significant increase in both IgA and IgG antibody titers in plasma of mice immunized with either RBD- or spike-adjuvanted formulations, while controls showed no responses (Figure 1D). Consistent with previous results, we observed comparable levels of RBD- and spike-directed IgG titers in mice immunized with adjuvanted RBD. While there was a greater titer of spike- compared to RBD-directed IgG responses in the AMVAD + spike formulation; boosting induced a greater proportion of RBD-specific antibodies in this group. In contrast, vaccination with CT + spike induced the greatest humoral responses of all vaccine combinations tested, consistent with previously reported results [41,43,48,60], with endpoint IgG titers near assay saturation (~5 × 10^6^) (Figure 1D).

**Figure 2:**
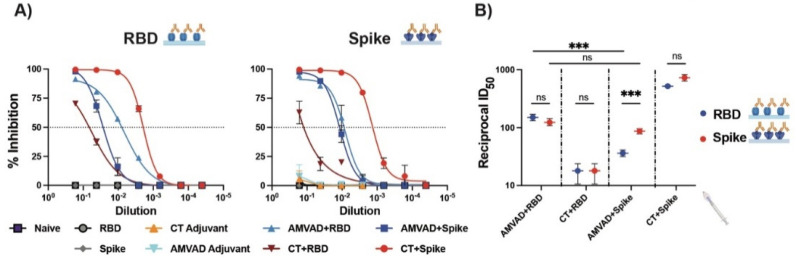
Systemic neutralizing antibodies induced by the intranasal administration with adjuvanted RBD or spike antigens. (**A**) Measurement of relative inhibition of soluble ACE2 and immobilized RBD or spike interaction by neutralizing antibodies in plasma collected at endpoint by surrogate neutralization ELISA (snELISA). (**B**) Reciprocal ID_50_ neutralizing titer of adjuvanted vaccine formulations in blocking RBD (blue) or spike (red) and ACE2 ligand interaction. This assay is representative of technical triplicates and presented as mean ± standard deviation. Statistical significance was calculated by one-way ANOVA followed by Tukey correction; n.s.: no statistical difference; *** *p* ≤ 0.001. Individual mice samples were pooled and analyzed in triplicate.

Induction of robust systemic humoral immunity, in particular neutralizing responses, has been shown to be a strong correlate of protection against severe disease [11,33,61,62]. Next, we functionally assessed the neutralizing responses of antibodies in plasma following the second immunization using a surrogate ELISA neutralization (snELISA) assay shown to quantitatively correlate with pseudotyped or infectious virus neutralization assay [57]. We evaluated the relative inhibition of ACE2–spike and ACE2–RBD ligand interactions and showed that neutralization responses followed the trends of systemic IgG titers (Figure 2A). Consistent with previous observations with antibody responses, we saw similar ID_50_ values of ACE2–RBD and ACE2–spike interactions with plasma of mice immunized with AMVAD + RBD (Figure 2B). In alignment with the ELISA results (Figure 1D), mice immunized with AMVAD + RBD showed significantly greater inhibition of ACE2–RBD than that observed with AMVAD + spike, thus suggesting that RBD immunogen focused antibody responses upon protective neutralizing epitopes localized within the RBD, preventing ACE2–RBD ligand interaction. In contrast, the ID_50_ of ACE2–spike interactions was comparable following immunizations with either AMVAD + RBD or AMVAD + spike, highlighting that neutralizing activity is not exclusively RBD directed. This is in line with other studies that suggest the influence of alternative neutralizing epitopes on the spike protein beyond the RBD, such as those in the N-terminal domain (Figure 2B) [18,33,63,64,65].

### 3.2. Adjuvanted Spike Antigen Immunization Induces Robust and Neutralizing Mucosal Responses in Mice

**Figure 3:**
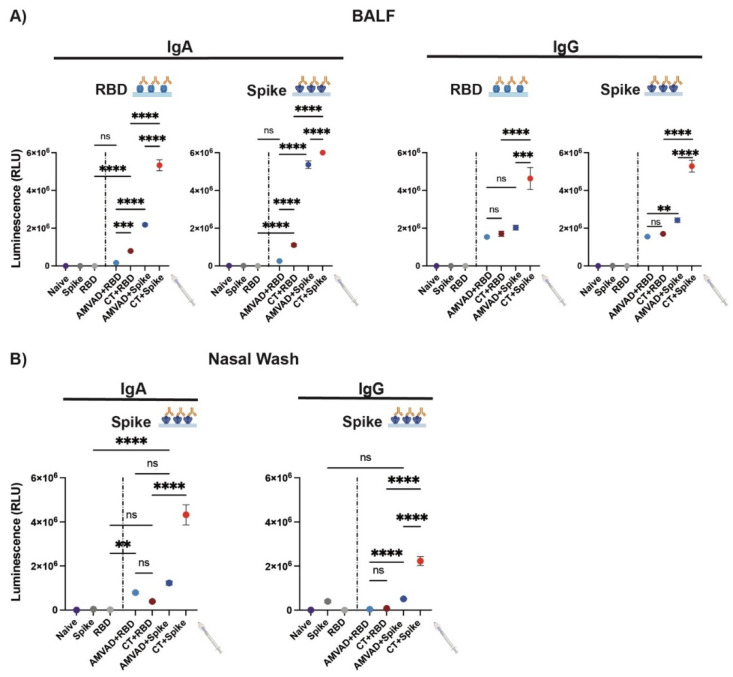
Antibody responses in BALF and the nasal cavity from intranasal administration with adjuvanted RBD or spike antigens. Measurement of RBD- and spike-specific IgA and IgG responses in (**A**) BALF and (**B**) nasal wash collected at end point. BALF was diluted 4- and 30-fold for measurement of IgA and IgG specific titers, respectively. Nasal wash was diluted four-fold. Statistical significance was calculated by one-way ANOVA followed by Tukey correction; n.s.: no statistical difference; ** *p* ≤ 0.01, *** *p* ≤ 0.001, **** *p* ≤ 0.0001. Individual mice samples were pooled and analyzed in triplicate.

Given the limited ability of currently available vaccines in inducing mucosal immunity, we set out to investigate mucosal humoral responses at the point of viral entry, the respiratory mucosa. To this end, we characterized IgA and IgG responses in BALF and nasal wash collected at the end point. We show that adjuvants are required for the induction of robust IgG and IgA responses (Figure 3). In particular, we observed significantly greater IgA and IgG titers in mice immunized with spike-adjuvanted formulations. Consistent with systemic antibody levels, AMVAD + spike-immunized mice showed robust spike- and RBD-specific IgA titers in BALF; however, AMVAD + RBD antibody levels were very low against both antigens at the experimental endpoint and did not reach statistical significance relative to RBD control (Figure 3A). While both of the RBD-adjuvanted formulations induced IgG responses against spike and RBD in BALF, only CT + RBD-vaccinated mice showed measurable IgA titers. Interestingly, the AMVAD + RBD formulation induced measurable IgA titers in the nasal wash that are comparable to AMVAD + spike (Figure 3B), and these are overall higher than IgG levels. This highlights the ability of intranasal adjuvated-antigen administration to more specifically induce IgA responses depending on the anatomical location within the mucosal compartment. Of note, the relative mucosal antibody titers measured in our study could be skewed given the high collection volume of buffer used during BALF and nasal wash sample acquisition relative to plasma collection.

### 3.3. Production of Neutralization Responses in Plasma of Immunized Mice against Ancestral and Variants of SARS-CoV-2

**Figure 4:**
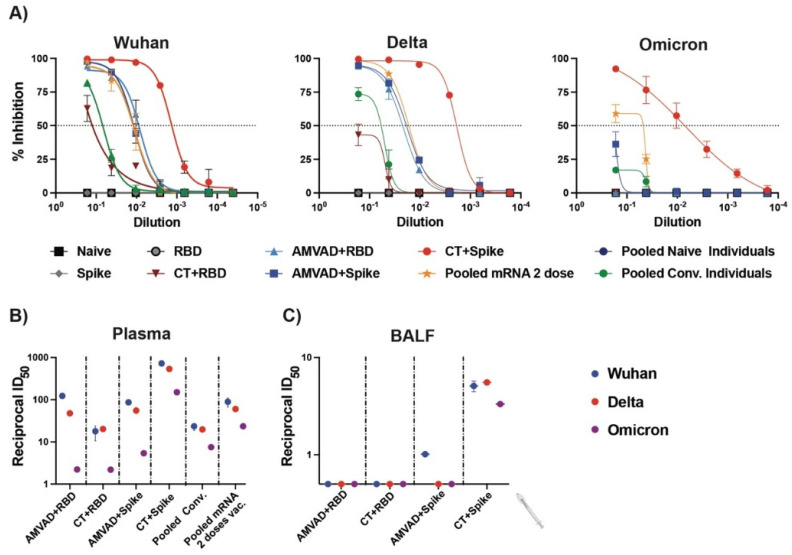
Neutralizing antibodies produced in plasma and BALF against SARS-CoV-2 variants. (**A**) Measurement of relative inhibition of ligand interaction between soluble ACE2 and Wuhan ancestral reference strain, Delta variant and Omicron variant spike protein by neutralizing antibodies in plasma at endpoint analyzed by snELISA. Reciprocal ID_50_ neutralizing titer of adjuvanted vaccine formulations in (**B**) plasma and (**C**) BALF blocking Wuhan reference strain (blue), Delta variant (red) and Omicron variant (purple) spike and ACE2 ligand interaction. This assay is representative of technical triplicates and presented as mean ± standard deviation.

Next, we compared the relative inhibition of ACE2–spike interactions by neutralizing antibodies in plasma and BALF for the ancestral, Delta and Omicron variants (Figure 4). We show that both adjuvanted RBD and spike formulations induced neutralizing antibodies in plasma against the spike proteins of Delta, but only CT–spike and, to a lesser extent, AMVAD + spike, induced the production of neutralizing antibodies against the spike of the Omicron variant (B.1.1.529) (Figure 4A,B). In particular, vaccination of mice with AMVAD-adjuvanted RBD or spike elicited superior inhibition of ACE2–spike interactions for Wuhan and Delta variants compared to natural SARS-CoV-2 infection in humans (i.e., pooled convalescent (pooled conv.); Alpha variant) and similar inhibition as pooled plasma from individuals vaccinated with two doses of the Pfizer mRNA vaccine (Figure 4A,B). Despite overall lower neutralization titers in BALF relative to levels observed in plasma, we showed that neutralization responses significantly correlated to IgA titers. Indeed, only spike-adjuvanted immunized mice showed weak but detectable neutralizing titers against SARS-CoV-2, with only CT + spike showing neutralizing activity against all the variants tested, while there was poor induction of mucosal neutralization with the adjuvanted RBD formulations (Figure 4C).

## 4. Discussion

Currently approved vaccines for SARS-CoV-2 induce robust humoral and cell-mediated immunity, which protect against severe disease, yet leave individuals vulnerable to infection at the URT where entry and replication first arise. Continued persistence of SARS-CoV-2, emergence of VOCs and poor induction of mucosal immunity contribute to decreased vaccine effectiveness against COVID-19 infection and onward transmission. Mucosal immunity is a critical component in the prevention and reduction of respiratory pathogen transmission [6,7]. Indeed, humoral responses in the mucosal compartment eliminate pathogens through various mechanisms, including blocking viral attachment to the cognate receptor via neutralizing antibodies, complement fixation and antibody-dependent cellular cytotoxicity [66,67]. As such, a growing body of work in the literature highlights the important role of mucosal secretory IgA in protecting against respiratory bacterial and viral pathogens that infect through the oral or nasal routes [7,19,20,21,23,26,27,28,29,30,66,67,68,69,70,71,72,73,74,75]. Stimulation of these humoral- and tissue-specific cell-mediated responses could be indispensable to blunt viral infection at the site of SARS-CoV-2 replication and limit viral transmission.

Since potent humoral responses have been shown to be a strong correlate of vaccine effectiveness and protection against COVID-19, we first analyzed mucosal and systemic IgG and IgA responses in mice vaccinated with spike and RBD adjuvanted with either CT or AMVAD. We show robust induction of IgG and IgA in plasma, nasal wash and BALF in mice vaccinated with two doses of adjuvanted protein vaccines, but not with protein or adjuvant alone. In line with previous reports, our data suggests that the combined administration of antigen with AMVAD likely led to its direct delivery to antigen-presenting cells for effective protein processing and presentation. Although the mechanism of action of AMVAD remains to be elucidated, its grape-like structure encompasses the antigen and acts as a delivery vehicle to elicit both humoral and cellular response [48,52]. Of note, we observed that vaccination with the RBD-adjuvanted vaccine favored RBD directed antibodies in both systemic and mucosal compartments, while immunization with adjuvanted spike induced broadly targeting antibodies that encompassed epitopes within and beyond the RBD domain. These findings were recapitulated when we evaluated relative inhibition of ACE2–spike and ACE2–RBD ligand interaction, where there was significantly lower inhibition of ACE2–RBD interaction in mice immunized with AMVAD + spike relative to AMVAD + RBD, yet comparable inhibition of ACE2–spike interaction between these two groups. Although immunization with RBD led to robust induction of systemic neutralizing antibodies blocking ACE2–RBD interactions for both ancestral and Delta variants, these neutralizing antibodies were poorly cross-reactive against the Omicron variant, which harbors more than 15 mutations within the RBD alone, suggesting that these antibodies likely targeted epitopes on the RBD prone to antigenic drift and escape by emerging VOCs [29,76,77,78,79]. In line with other comparative protein subunit antigen immunogenicity studies via intramuscular vaccination [1,53,80,81], our findings suggest that intranasal immunization with adjuvanted spike formulation induced greater breadth of neutralizing antibodies that target both conserved and variable epitopes of the spike. In addition, trimeric spike has been shown to be more immunogenic given the larger T cell epitope repertoire leading to a greater recruitment of T helper follicular cells and, thereby, an enhanced humoral response relative to the RBD antigen, as shown by Tan et al. [80]. Indeed, non-RBD directed antibodies have been shown to be important for both mucosal and systemic humoral immunity in preventing severe disease from both SARS-CoV-1 and SARS-CoV-2 infection [64,82,83].

In plasma, we observed that high levels of IgG correlated with increased neutralization titers, while in BALF, potent IgA levels correlated with neutralizing responses as observed in previous influenza and SARS-CoV-2 vaccination studies [29,66,75,84]. Notably, AMVAD + spike formulation induced significantly greater IgA levels in both plasma and BALF relative to AMVAD + RBD immunization. Given that RBD + AMVAD-immunized mice showed low levels of IgA and undetectable neutralizing activity in BALF, we speculate that mucosal IgA antibodies largely contributed to ACE2–spike inhibition observed in spike-adjuvanted groups. Thus, the spike’s superior immunogenicity could also be attributed to its larger and stabilized trimeric structure compared to RBD’s monomer, where the spike protein’s repetitive trimeric antigen presentation could increase cross-linking to B cell receptors, inducing greater B cell activation and IgA response [85,86].

Induction of protective humoral immunity has been a major focus in COVID-19 vaccine development. Nevertheless, robust induction of secretory IgA antibodies is crucial given their potent and broadly neutralizing capabilities [20,29,66,67,68,69,71,87,88]. Studies of intranasal vaccines against influenza have highlighted that mucosal vaccination, compared to the intramuscular route, elicited potent cross-protection against heterosubtypic challenge of influenza and showed long-term immunity [66,89]. Additionally, in a proof-of-concept study, Xiao et al. demonstrated that intranasal immunization with defective viral particles provides powerful prophylactic, broad-spectrum protection from infection by SARS-CoV-2 and influenza virus by blocking viral replication and preventing reinfection [90]. This is further reiterated by several groups that showed superior protection with intranasal relative to intramuscular immunization in mice against challenge with ancestral SARS-CoV-2 strain and VOCs [19,20,21,23,24,30]. Indeed, Langel et al., demonstrated reduced SARS-CoV-2 airborne transmission in intranasally immunized hamsters [21]. In addition, Tang et al. demonstrated that convalescent individuals show increased levels of broadly neutralizing antibodies in BALF relative to vaccinated individuals, despite similar systemic IgG titers [29]. Epidemiological surveillance studies highlight the important role that mucosal IgA may have in preventing SARS-CoV-2 infection. In fact, Sheikh et al. and Havervall et al. demonstrated that vaccinated participants who experienced breakthrough infections with divergent VOCs had lower titers of serum and mucosal IgA relative to those who did not experience an infection despite having comparable serum IgG titers [72,73]. Here, we showed an adjuvanted subunit vaccine platform that induced robust mucosal and systemic total and neutralizing antibody responses against rapidly emerging SARS-CoV-2 variants. In our direct comparison of the immunogenic profiles of SARS-CoV-2 spike and RBD antigens, we highlight the relative merit of additional epitopes of the larger spike protein that improve protein immunogenicity at the respiratory mucosa and neutralizing activity against VOCs. Continued SARS-CoV-2 transmission and replication, and selection pressure induced by vaccination or prior infection has led to the emergence of highly transmissible VOCs. Given the persistence of the SARS-CoV-2 pandemic and the shortcomings of currently available vaccines in effectively blocking transmission, our study may offer insights for selecting immunogenic targets for intranasal vaccine design against current and future VOCs. Thus, new modalities of vaccination that invoke mucosal immunity are crucial to neutralize the virus, limit the initial infection at the point of entry, prevent onward transmission and curtail the COVID-19 pandemic.

## Data Availability

The data presented in this study are available on request from the corresponding author. The data are not publicly available due to confidentiality issues with interviewees.

## References

[1] Cele S., Jackson L., Khoury D.S., Khan K., Moyo-Gwete T., Tegally H., San J.E., Cromer D., Scheepers C., Amoako D. (2021). SARS-CoV-2 Omicron has extensive but incomplete escape of Pfizer BNT162b2 elicited neutralization and requires ACE2 for infection. medRxiv.

[2] Chemaitelly H., Tang P., Hasan M.R., AlMukdad S., Yassine H.M., Benslimane F.M., Al Khatib H.A., Coyle P., Ayoub H.H., Al Kanaani Z. (2021). Waning of BNT162b2 Vaccine Protection against SARS-CoV-2 Infection in Qatar. N. Engl. J. Med..

[3] Greaney A.J., Starr T.N., Gilchuk P., Zost S.J., Binshtein E., Loes A.N., Hilton S.K., Huddleston J., Eguia R., Crawford K.H.D. (2021). Complete Mapping of Mutations to the SARS-CoV-2 Spike Receptor-Binding Domain that Escape Antibody Recognition. Cell Host Microbe.

[4] Menni C., May A., Polidori L., Louca P., Wolf J., Capdevila J., Hu C., Ourselin S., Steves C.J., Valdes A.M. (2022). COVID-19 vaccine waning and effectiveness and side-effects of boosters: A prospective community study from the ZOE COVID Study. Lancet Infect. Dis..

[5] Singanayagam A., Hakki S., Dunning J., Madon K.J., Crone M.A., Koycheva A., Derqui-Fernandez N., Barnett J.L., Whitfield M.G., Varro R. (2022). Community transmission and viral load kinetics of the SARS-CoV-2 delta (B.1.617.2) variant in vaccinated and unvaccinated individuals in the UK: A prospective, longitudinal, cohort study. Lancet Infect. Dis..

[6] Krammer F. (2020). SARS-CoV-2 vaccines in development. Nature.

[7] Lavelle E.C., Ward R.W. (2022). Mucosal vaccines—Fortifying the frontiers. Nat. Rev. Immunol..

[8] Kemp S.A., Cheng M.T.K., Hamilton W.L., Kamelian K., Indian S.-C.-G.C., Singh S., Rakshit P., Agrawal A., Illingworth C.J.R., Gupta R.K. (2022). Transmission of B.1.617.2 Delta variant between vaccinated healthcare workers. Sci. Rep..

[9] Chia P.Y., Ong S.W.X., Chiew C.J., Ang L.W., Chavatte J.M., Mak T.M., Cui L., Kalimuddin S., Chia W.N., Tan C.W. (2022). Virological and serological kinetics of SARS-CoV-2 Delta variant vaccine breakthrough infections: A multicentre cohort study. Clin. Microbiol. Infect..

[10] Lange B., Gerigk M., Tenenbaum T. (2021). Breakthrough Infections in BNT162b2-Vaccinated Health Care Workers. N. Engl. J. Med..

[11] Bartsch Y.C., Fischinger S., Siddiqui S.M., Chen Z., Yu J., Gebre M., Atyeo C., Gorman M.J., Zhu A.L., Kang J. (2021). Discrete SARS-CoV-2 antibody titers track with functional humoral stability. Nat. Commun..

[12] Bartsch Y.C., Wang C., Zohar T., Fischinger S., Atyeo C., Burke J.S., Kang J., Edlow A.G., Fasano A., Baden L.R. (2021). Humoral signatures of protective and pathological SARS-CoV-2 infection in children. Nat. Med..

[13] Brouwer P.J.M., Caniels T.G., van der Straten K., Snitselaar J.L., Aldon Y., Bangaru S., Torres J.L., Okba N.M.A., Claireaux M., Kerster G. (2020). Potent neutralizing antibodies from COVID-19 patients define multiple targets of vulnerability. Science.

[14] Cao Y., Su B., Guo X., Sun W., Deng Y., Bao L., Zhu Q., Zhang X., Zheng Y., Geng C. (2020). Potent Neutralizing Antibodies against SARS-CoV-2 Identified by High-Throughput Single-Cell Sequencing of Convalescent Patients’ B Cells. Cell.

[15] Kreer C., Zehner M., Weber T., Ercanoglu M.S., Gieselmann L., Rohde C., Halwe S., Korenkov M., Schommers P., Vanshylla K. (2020). Longitudinal Isolation of Potent Near-Germline SARS-CoV-2-Neutralizing Antibodies from COVID-19 Patients. Cell.

[16] Rogers T.F., Zhao F., Huang D., Beutler N., Burns A., He W.T., Limbo O., Smith C., Song G., Woehl J. (2020). Isolation of potent SARS-CoV-2 neutralizing antibodies and protection from disease in a small animal model. Science.

[17] Zost S.J., Gilchuk P., Case J.B., Binshtein E., Chen R.E., Nkolola J.P., Schafer A., Reidy J.X., Trivette A., Nargi R.S. (2020). Potently neutralizing and protective human antibodies against SARS-CoV-2. Nature.

[18] Liu L., Wang P., Nair M.S., Yu J., Rapp M., Wang Q., Luo Y., Chan J.F., Sahi V., Figueroa A. (2020). Potent neutralizing antibodies against multiple epitopes on SARS-CoV-2 spike. Nature.

[19] Afkhami S., D’Agostino M.R., Zhang A., Stacey H.D., Marzok A., Kang A., Singh R., Bavananthasivam J., Ye G., Luo X. (2022). Respiratory mucosal delivery of next-generation COVID-19 vaccine provides robust protection against both ancestral and variant strains of SARS-CoV-2. Cell.

[20] Mao T., Israelow B., Suberi A., Zhou L., Reschke M., Pena-Hernandez M.A., Dong H., Homer R.J., Saltzman W.M., Iwasaki A. (2022). Unadjuvanted intranasal spike vaccine booster elicits robust protective mucosal immunity against sarbecoviruses. Science.

[21] Langel S.N., Johnson S., Martinez C.I., Tedjakusuma S.N., Peinovich N., Dora E.G., Kuehl P.J., Irshad H., Barrett E.G., Werts A. (2022). Adenovirus type 5 SARS-CoV-2 vaccines delivered orally or intranasally reduced disease severity and transmission in a hamster model. Sci. Transl. Med..

[22] Diaz M.F., Calderon K., Rojas-Neyra A., Vakharia V.N., Choque-Guevara R., Montalvan-Avalos A., Poma-Acevedo A., Rios-Matos D., Agurto-Arteaga A., Cauti-Mendoza M.G. (2022). Intranasal vaccination of hamsters with a Newcastle disease virus vector expressing the S1 subunit protects animals against SARS-CoV-2 disease. Sci. Rep..

[23] Hassan A.O., Feldmann F., Zhao H., Curiel D.T., Okumura A., Tang-Huau T.L., Case J.B., Meade-White K., Callison J., Lovaglio J. (2021). A single intranasal dose of chimpanzee adenovirus-vectored vaccine protects against SARS-CoV-2 infection in rhesus macaques. bioRxiv.

[24] Bricker T.L., Darling T.L., Hassan A.O., Harastani H.H., Soung A., Jiang X., Dai Y.N., Zhao H., Adams L.J., Holtzman M.J. (2021). A single intranasal or intramuscular immunization with chimpanzee adenovirus-vectored SARS-CoV-2 vaccine protects against pneumonia in hamsters. Cell Rep..

[25] van Doremalen N., Purushotham J.N., Schulz J.E., Holbrook M.G., Bushmaker T., Carmody A., Port J.R., Yinda C.K., Okumura A., Saturday G. (2021). Intranasal ChAdOx1 nCoV-19/AZD1222 vaccination reduces viral shedding after SARS-CoV-2 D614G challenge in preclinical models. Sci. Transl. Med..

[26] King R.G., Silva-Sanchez A., Peel J.N., Botta D., Dickson A.M., Pinto A.K., Meza-Perez S., Allie S.R., Schultz M.D., Liu M. (2021). Single-Dose Intranasal Administration of AdCOVID Elicits Systemic and Mucosal Immunity against SARS-CoV-2 and Fully Protects Mice from Lethal Challenge. Vaccines.

[27] An X., Martinez-Paniagua M., Rezvan A., Sefat S.R., Fathi M., Singh S., Biswas S., Pourpak M., Yee C., Liu X. (2021). Single-dose intranasal vaccination elicits systemic and mucosal immunity against SARS-CoV-2. iScience.

[28] Du Y., Xu Y., Feng J., Hu L., Zhang Y., Zhang B., Guo W., Mai R., Chen L., Fang J. (2021). Intranasal administration of a recombinant RBD vaccine induced protective immunity against SARS-CoV-2 in mouse. Vaccine.

[29] Tang J., Zeng C., Cox T.M., Li C., Son Y.M., Cheon I.S., Wu Y., Behl S., Taylor J.J., Chakraborty R. (2022). Respiratory mucosal immunity against SARS-CoV-2 following mRNA vaccination. Sci. Immunol..

[30] Stark F.C., Akache B., Deschatelets L., Tran A., Stuible M., Durocher Y., McCluskie M.J., Agbayani G., Dudani R., Harrison B.A. (2022). Intranasal immunization with a proteosome-adjuvanted SARS-CoV-2 spike protein-based vaccine is immunogenic and efficacious in mice and hamsters. Sci. Rep..

[31] Hassan A.O., Kafai N.M., Dmitriev I.P., Fox J.M., Smith B.K., Harvey I.B., Chen R.E., Winkler E.S., Wessel A.W., Case J.B. (2020). A Single-Dose Intranasal ChAd Vaccine Protects Upper and Lower Respiratory Tracts against SARS-CoV-2. Cell.

[32] Kleanthous H., Silverman J.M., Makar K.W., Yoon I.K., Jackson N., Vaughn D.W. (2021). Scientific rationale for developing potent RBD-based vaccines targeting COVID-19. npj Vaccines.

[33] Garcia-Beltran W.F., Lam E.C., Astudillo M.G., Yang D., Miller T.E., Feldman J., Hauser B.M., Caradonna T.M., Clayton K.L., Nitido A.D. (2021). COVID-19-neutralizing antibodies predict disease severity and survival. Cell.

[34] Pinto D., Park Y.J., Beltramello M., Walls A.C., Tortorici M.A., Bianchi S., Jaconi S., Culap K., Zatta F., De Marco A. (2020). Cross-neutralization of SARS-CoV-2 by a human monoclonal SARS-CoV antibody. Nature.

[35] Barnes C.O., Jette C.A., Abernathy M.E., Dam K.A., Esswein S.R., Gristick H.B., Malyutin A.G., Sharaf N.G., Huey-Tubman K.E., Lee Y.E. (2020). SARS-CoV-2 neutralizing antibody structures inform therapeutic strategies. Nature.

[36] Piccoli L., Park Y.J., Tortorici M.A., Czudnochowski N., Walls A.C., Beltramello M., Silacci-Fregni C., Pinto D., Rosen L.E., Bowen J.E. (2020). Mapping Neutralizing and Immunodominant Sites on the SARS-CoV-2 Spike Receptor-Binding Domain by Structure-Guided High-Resolution Serology. Cell.

[37] Liang Z., Zhu H., Wang X., Jing B., Li Z., Xia X., Sun H., Yang Y., Zhang W., Shi L. (2020). Adjuvants for Coronavirus Vaccines. Front. Immunol..

[38] Fujkuyama Y., Tokuhara D., Kataoka K., Gilbert R.S., McGhee J.R., Yuki Y., Kiyono H., Fujihashi K. (2012). Novel vaccine development strategies for inducing mucosal immunity. Expert Rev. Vaccines.

[39] Chugh Y., Kapoor P., Kapoor A.K. (2009). Intranasal drug delivery: A novel approach. Indian J. Otolaryngol. Head Neck Surg..

[40] Wang Y.Y., Lai S.K., So C., Schneider C., Cone R., Hanes J. (2011). Mucoadhesive nanoparticles may disrupt the protective human mucus barrier by altering its microstructure. PLoS ONE.

[41] McGee D.W., Elson C.O., McGhee J.R. (1993). Enhancing effect of cholera toxin on interleukin-6 secretion by IEC-6 intestinal epithelial cells: Mode of action and augmenting effect of inflammatory cytokines. Infect. Immun..

[42] Lucas C., Vogels C.B.F., Yildirim I., Rothman J.E., Lu P., Monteiro V., Gehlhausen J.R., Campbell M., Silva J., Tabachnikova A. (2021). Impact of circulating SARS-CoV-2 variants on mRNA vaccine-induced immunity. Nature.

[43] Lycke N., Bemark M. (2010). Mucosal adjuvants and long-term memory development with special focus on CTA1-DD and other ADP-ribosylating toxins. Mucosal Immunol..

[44] Kim Y.G. (2017). Microbiota Influences Vaccine and Mucosal Adjuvant Efficacy. Immune Netw..

[45] Clements J.D., Norton E.B. (2018). The Mucosal Vaccine Adjuvant LT(R192G/L211A) or dmLT. mSphere.

[46] Lewis D.J., Huo Z., Barnett S., Kromann I., Giemza R., Galiza E., Woodrow M., Thierry-Carstensen B., Andersen P., Novicki D. (2009). Transient facial nerve paralysis (Bell’s palsy) following intranasal delivery of a genetically detoxified mutant of *Escherichia coli* heat labile toxin. PLoS ONE.

[47] Mutsch M., Zhou W., Rhodes P., Bopp M., Chen R.T., Linder T., Spyr C., Steffen R. (2004). Use of the inactivated intranasal influenza vaccine and the risk of Bell’s palsy in Switzerland. N. Engl. J. Med..

[48] Patel G.B., Zhou H., Ponce A., Chen W. (2007). Mucosal and systemic immune responses by intranasal immunization using archaeal lipid-adjuvanted vaccines. Vaccine.

[49] Patel G.B., Zhou H., Ponce A., Harris G., Chen W. (2010). Intranasal immunization with an archaeal lipid mucosal vaccine adjuvant and delivery formulation protects against a respiratory pathogen challenge. PLoS ONE.

[50] Patel G.B., Chen W. (2010). Archaeal lipid mucosal vaccine adjuvant and delivery system. Expert Rev. Vaccines.

[51] Patel G.B., Ponce A., Zhou H., Chen W. (2008). Safety of intranasally administered archaeal lipid mucosal vaccine adjuvant and delivery (AMVAD) vaccine in mice. Int. J. Toxicol..

[52] Patel G.B., Ponce A., Zhou H., Chen W. (2008). Structural characterization of archaeal lipid mucosal vaccine adjuvant and delivery (AMVAD) formulations prepared by different protocols and their efficacy upon intranasal immunization of mice. J. Liposome Res..

[53] Akache B., Renner T.M., Tran A., Deschatelets L., Dudani R., Harrison B.A., Duque D., Haukenfrers J., Rossotti M.A., Gaudreault F. (2021). Immunogenic and efficacious SARS-CoV-2 vaccine based on resistin-trimerized spike antigen SmT1 and SLA archaeosome adjuvant. Sci. Rep..

[54] Akache B., Deschatelets L., Harrison B.A., Dudani R., Stark F.C., Jia Y., Landi A., Law J.L.M., Logan M., Hockman D. (2019). Effect of Different Adjuvants on the Longevity and Strength of Humoral and Cellular Immune Responses to the HCV Envelope Glycoproteins. Vaccines.

[55] Krishnan L., Sad S., Patel G.B., Sprott G.D. (2001). The potent adjuvant activity of archaeosomes correlates to the recruitment and activation of macrophages and dendritic cells In Vivo. J. Immunol..

[56] Krishnan L., Dicaire C.J., Patel G.B., Sprott G.D. (2000). Archaeosome vaccine adjuvants induce strong humoral, cell-mediated, and memory responses: Comparison to conventional liposomes and alum. Infect. Immun..

[57] Colwill K., Galipeau Y., Stuible M., Gervais C., Arnold C., Rathod B., Abe K.T., Wang J.H., Pasculescu A., Maltseva M. (2022). A scalable serology solution for profiling humoral immune responses to SARS-CoV-2 infection and vaccination. Clin. Transl. Immunol..

[58] Demone J., Maltseva M., Nourimand M., Nasr-Sharif M., Galipeau Y., Alarcon E.I., Langlois M.A., MacLean A.M. (2022). Scalable agroinfiltration-based production of SARS-CoV-2 antigens for use in diagnostic assays and subunit vaccines. PLoS ONE.

[59] Abe K.T., Li Z., Samson R., Samavarchi-Tehrani P., Valcourt E.J., Wood H., Budylowski P., Dupuis A.P., Girardin R.C., Rathod B. (2020). A simple protein-based surrogate neutralization assay for SARS-CoV-2. JCI Insight.

[60] Mattsson J., Schon K., Ekman L., Fahlen-Yrlid L., Yrlid U., Lycke N.Y. (2015). Cholera toxin adjuvant promotes a balanced Th1/Th2/Th17 response independently of IL-12 and IL-17 by acting on Gsalpha in CD11b^+^ DCs. Mucosal Immunol..

[61] Cromer D., Steain M., Reynaldi A., Schlub T.E., Wheatley A.K., Juno J.A., Kent S.J., Triccas J.A., Khoury D.S., Davenport M.P. (2022). Neutralising antibody titres as predictors of protection against SARS-CoV-2 variants and the impact of boosting: A meta-analysis. Lancet Microbe.

[62] Khoury D.S., Cromer D., Reynaldi A., Schlub T.E., Wheatley A.K., Juno J.A., Subbarao K., Kent S.J., Triccas J.A., Davenport M.P. (2021). Neutralizing antibody levels are highly predictive of immune protection from symptomatic SARS-CoV-2 infection. Nat. Med..

[63] Li T., Han X., Gu C., Guo H., Zhang H., Wang Y., Hu C., Wang K., Liu F., Luo F. (2021). Potent SARS-CoV-2 neutralizing antibodies with protective efficacy against newly emerged mutational variants. Nat. Commun..

[64] Ng K.W., Faulkner N., Finsterbusch K., Wu M., Harvey R., Hussain S., Greco M., Liu Y., Kjaer S., Swanton C. (2022). SARS-CoV-2 S2-targeted vaccination elicits broadly neutralizing antibodies. Sci. Transl. Med..

[65] Chi X., Yan R., Zhang J., Zhang G., Zhang Y., Hao M., Zhang Z., Fan P., Dong Y., Yang Y. (2020). A neutralizing human antibody binds to the N-terminal domain of the Spike protein of SARS-CoV-2. Science.

[66] Oh J.E., Song E., Moriyama M., Wong P., Zhang S., Jiang R., Strohmeier S., Kleinstein S.H., Krammer F., Iwasaki A. (2021). Intranasal priming induces local lung-resident B cell populations that secrete protective mucosal antiviral IgA. Sci. Immunol..

[67] Iwasaki A. (2016). Exploiting Mucosal Immunity for Antiviral Vaccines. Annu. Rev. Immunol..

[68] Lindley R.A., Steele E.J. (2021). Analysis of SARS-CoV-2 haplotypes and genomic sequences during 2020 in Victoria, Australia, in the context of putative deficits in innate immune deaminase anti-viral responses. Scand. J. Immunol..

[69] Gorczynski R.M., Lindley R.A., Steele E.J., Wickramasinghe N.C. (2021). Nature of Acquired Immune Responses, Epitope Specificity and Resultant Protection from SARS-CoV-2. J. Pers. Med..

[70] Wilkie B.N. (1982). Respiratory tract immune response to microbial pathogens. J. Am. Vet. Med. Assoc..

[71] Sheikh-Mohamed S., Sanders E.C., Gommerman J.L., Tal M.C. (2022). Guardians of the oral and nasopharyngeal galaxy: IgA and protection against SARS-CoV-2 infection. Immunol. Rev..

[72] Sheikh-Mohamed S., Isho B., Chao G.Y.C., Zuo M., Cohen C., Lustig Y., Nahass G.R., Salomon-Shulman R.E., Blacker G., Fazel-Zarandi M. (2022). Systemic and mucosal IgA responses are variably induced in response to SARS-CoV-2 mRNA vaccination and are associated with protection against subsequent infection. Mucosal Immunol..

[73] Havervall S., Marking U., Svensson J., Greilert-Norin N., Bacchus P., Nilsson P., Hober S., Gordon M., Blom K., Klingstrom J. (2022). Anti-Spike Mucosal IgA Protection against SARS-CoV-2 Omicron Infection. N. Engl. J. Med..

[74] Cervia C., Nilsson J., Zurbuchen Y., Valaperti A., Schreiner J., Wolfensberger A., Raeber M.E., Adamo S., Weigang S., Emmenegger M. (2021). Systemic and mucosal antibody responses specific to SARS-CoV-2 during mild versus severe COVID-19. J. Allergy Clin. Immunol..

[75] Sterlin D., Mathian A., Miyara M., Mohr A., Anna F., Claer L., Quentric P., Fadlallah J., Devilliers H., Ghillani P. (2021). IgA dominates the early neutralizing antibody response to SARS-CoV-2. Sci. Transl. Med..

[76] Schmidt F., Muecksch F., Weisblum Y., Da Silva J., Bednarski E., Cho A., Wang Z., Gaebler C., Caskey M., Nussenzweig M.C. (2022). Plasma Neutralization of the SARS-CoV-2 Omicron Variant. N. Engl. J. Med..

[77] Burki T.K. (2022). Omicron variant and booster COVID-19 vaccines. Lancet Respir. Med..

[78] Liu L., Iketani S., Guo Y., Chan J.F., Wang M., Liu L., Luo Y., Chu H., Huang Y., Nair M.S. (2022). Striking antibody evasion manifested by the Omicron variant of SARS-CoV-2. Nature.

[79] Yamasoba D., Kimura I., Nasser H., Morioka Y., Nao N., Ito J., Uriu K., Tsuda M., Zahradnik J., Shirakawa K. (2022). Virological characteristics of the SARS-CoV-2 Omicron BA.2 spike. Cell.

[80] Tan H.X., Juno J.A., Lee W.S., Barber-Axthelm I., Kelly H.G., Wragg K.M., Esterbauer R., Amarasena T., Mordant F.L., Subbarao K. (2021). Immunogenicity of prime-boost protein subunit vaccine strategies against SARS-CoV-2 in mice and macaques. Nat. Commun..

[81] Mandolesi M., Sheward D.J., Hanke L., Ma J., Pushparaj P., Perez Vidakovics L., Kim C., Adori M., Lenart K., Lore K. (2021). SARS-CoV-2 protein subunit vaccination of mice and rhesus macaques elicits potent and durable neutralizing antibody responses. Cell Rep. Med..

[82] Zhong X., Yang H., Guo Z.F., Sin W.Y., Chen W., Xu J., Fu L., Wu J., Mak C.K., Cheng C.S. (2005). B-cell responses in patients who have recovered from severe acute respiratory syndrome target a dominant site in the S2 domain of the surface spike glycoprotein. J. Virol..

[83] Zohar T., Loos C., Fischinger S., Atyeo C., Wang C., Slein M.D., Burke J., Yu J., Feldman J., Hauser B.M. (2020). Compromised Humoral Functional Evolution Tracks with SARS-CoV-2 Mortality. Cell.

[84] Butler S.E., Crowley A.R., Natarajan H., Xu S., Weiner J.A., Bobak C.A., Mattox D.E., Lee J., Wieland-Alter W., Connor R.I. (2020). Distinct Features and Functions of Systemic and Mucosal Humoral Immunity Among SARS-CoV-2 Convalescent Individuals. Front. Immunol..

[85] Bachmann M.F., Zinkernagel R.M. (1997). Neutralizing antiviral B cell responses. Annu. Rev. Immunol..

[86] Bachmann M.F., Jennings G.T. (2010). Vaccine delivery: A matter of size, geometry, kinetics and molecular patterns. Nat. Rev. Immunol..

[87] Suzuki T., Kawaguchi A., Ainai A., Tamura S., Ito R., Multihartina P., Setiawaty V., Pangesti K.N., Odagiri T., Tashiro M. (2015). Relationship of the quaternary structure of human secretory IgA to neutralization of influenza virus. Proc. Natl. Acad. Sci. USA.

[88] Zeng W., Ma H., Ding C., Yang Y., Sun Y., Huang X., He W., Xiang Y., Gao Y., Jin T. (2021). Characterization of SARS-CoV-2-specific antibodies in COVID-19 patients reveals highly potent neutralizing IgA. Signal Transduct. Target. Ther..

[89] Zens K.D., Chen J.K., Farber D.L. (2016). Vaccine-generated lung tissue-resident memory T cells provide heterosubtypic protection to influenza infection. JCI Insight.

[90] Xiao Y., Lidsky P.V., Shirogane Y., Aviner R., Wu C.T., Li W., Zheng W., Talbot D., Catching A., Doitsh G. (2021). A defective viral genome strategy elicits broad protective immunity against respiratory viruses. Cell.

